# Supplementary material for: Time-series transcriptome analysis identified differentially expressed genes in broiler chicken infected with mixed Eimeria species
Source: Front Genet. 2022 Aug 8;13:886781. doi: 10.3389/fgene.2022.886781 (PMC9393255; doi:10.3389/fgene.2022.886781)
Supplement: Supplementary file 2 [file DataSheet1.ZIP › 7dpi_KEGG.Gsea.1625115961950/KEGG_TERPENOID_BACKBONE_BIOSYNTHESIS.html]

Details for gene set KEGG\_TERPENOID\_BACKBONE\_BIOSYNTHESIS[GSEA]

|  || Dataset | TMM\_7dpi\_gct\_format\_7dpi\_gct\_format.Class\_4dpi.cls #PC\_versus\_NC.Class\_4dpi.cls #PC\_versus\_NC\_repos |
| Phenotype | Class\_4dpi.cls#PC\_versus\_NC\_repos |
| Upregulated in class | 1 |
| GeneSet | KEGG\_TERPENOID\_BACKBONE\_BIOSYNTHESIS |
| Enrichment Score (ES) | 0.47875538 |
| Normalized Enrichment Score (NES) | 1.4307815 |
| Nominal p-value | 0.08695652 |
| FDR q-value | 0.19965741 |
| FWER p-Value | 0.807 |
Table: GSEA Results Summary

  

Fig 1: Enrichment plot: KEGG\_TERPENOID\_BACKBONE\_BIOSYNTHESIS      
 Profile of the Running ES Score & Positions of GeneSet Members on the Rank Ordered List

  

| SYMBOL | TITLE | RANK IN GENE LIST | RANK METRIC SCORE | RUNNING ES | CORE ENRICHMENT || 1 | PMVK | na | 373 | 0.856 | 0.1559 | Yes |
| 2 | MVD | na | 636 | 0.669 | 0.2803 | Yes |
| 3 | FDPS | na | 714 | 0.629 | 0.4114 | Yes |
| 4 | ACAT1 | na | 1144 | 0.472 | 0.4788 | Yes |
| 5 | GGPS1 | na | 2387 | 0.209 | 0.4210 | No |
| 6 | ACAT2 | na | 3304 | 0.086 | 0.3634 | No |
| 7 | PDSS1 | na | 4563 | -0.057 | 0.2711 | No |
| 8 | IDI2 | na | 5580 | -0.156 | 0.2206 | No |
| 9 | HMGCR | na | 6636 | -0.269 | 0.1914 | No |
| 10 | PDSS2 | na | 6955 | -0.303 | 0.2311 | No |
| 11 | MVK | na | 7347 | -0.346 | 0.2742 | No |
| 12 | HMGCS1 | na | 8787 | -0.523 | 0.2686 | No |
Table: GSEA details [plain text format]

  

Fig 2: KEGG\_TERPENOID\_BACKBONE\_BIOSYNTHESIS      
 Blue-Pink O' Gram in the Space of the Analyzed GeneSet

  

Fig 3: KEGG\_TERPENOID\_BACKBONE\_BIOSYNTHESIS: Random ES distribution      
 Gene set null distribution of ES for **KEGG\_TERPENOID\_BACKBONE\_BIOSYNTHESIS**
